# CAPN1 promotes malignant behavior and erlotinib resistance mediated by phosphorylation of c‐Met and PIK3R2 *via* degrading PTPN1 in lung adenocarcinoma

**DOI:** 10.1111/1759-7714.13465

**Published:** 2020-05-12

**Authors:** Yichuan Chen, Jingqun Tang, Ting Lu, Fang Liu

**Affiliations:** ^1^ Department of Cardiovascular Surgery, The Second Xiangya Hospital Central South University Changsha China; ^2^ Department of Thoracic Surgery, The Second Xiangya Hospital Central South University Changsha China; ^3^ Clinic Nursing Teaching and Research Section, The Second Xiangya Hospital Central South University Changsha China

**Keywords:** Calpain 1, drug resistance, EGFR‐TKI, lung adenocarcinoma, protein tyrosine phosphatase, non‐receptor type 1

## Abstract

**Background:**

Calpain 1 (CAPN1) has been found to be a promoter of cancer progression. PTPN1 as a physiological target molecule of CAPN1 plays a dephosphorylated role on multiple receptor tyrosine kinases. This study aimed to reveal the effects of CAPN1/PTPN1 on malignant phenotype and EGFR‐TKI resistance of lung adenocarcinoma (LUAD) cells.

**Methods:**

A total of 84 primary LUAD tissues and paired paracancerous normal tissues were collected. Quantitative real‐time PCR (qRT‐PCR) and immunohistochemical (IHC) methods were used to measure the expression of CAPN1 and PTPN1 in tissues. qRT‐PCR and western blot were used to detect the expressions of CAPN1, PTPN1, c‐Met and PIK3R2 in cell lines. Cell counting kit‐8 (CCK‐8), colony formation and transwell assay were carried out to evaluate cell erlotinib resistance, proliferation, migration and invasion. Co‐IP assay was used to verify the interaction between proteins. Cycloheximide (CHX) was applied to block protein synthesis.

**Results:**

CAPN1, c‐Met and PIK3R2 were significantly upregulated and the correlation was positive in LUAD, while PTPN1 was decreased. *EGFR*‐sensitive mutation was related to CAPN1/PTPN1. in vitro studies showed that PTPN1 can mediate dephosphorylation of c‐Met and PIK3R2 by binding with both, thereby weakening cell proliferation, metastasis and erlotinib resistance, while CAPN1 could enhance the degradation of PTPN1 protein as a cancer promoter.

**Conclusions:**

CAPN1 enhances the malignant behavior and erlotinib resistance of LUAD cells via degrading PTPN1 and then activating c‐Met/PIK3R2, which suggests CAPN1/PTPN1 may serve as tumor markers or potential targets for diagnosis and treatment of LUAD.

**Key points:**

**Significant findings of the study**

Superior CAPN1 and inferior PTPN1 were related to activation of c‐Met/PIK3R2 in lung adenocarcinoma. Moreover, regulations of CAPN1 and PTPN1 induced the changes of malignant behavior and erlotinib resistance.

**What this study adds**

Our findings confirmed that CAPN1/PTPN1 play crucial roles on proliferation, metastasis and erlotinib resistance of LUAD cells as c‐Met/PIK3R2 regulators, and validated the regulatory mechanism of CAPN1 on PTPN1 in tumor model for the first time.

## Introduction

According to the data of the American Cancer Association, 2.09 million new cases and 1.76 million deaths of patients with lung cancer occurred in the world in 2018, ranking first in both incidence and mortality.^1^ Lung adenocarcinoma (LUAD) is the main pathological type of lung cancer, whose occurrence and development involves serial activation of pro‐oncogenes and inactivation of suppressor genes. In a prospective cohort of 1450 Asian patients, 51.4% were confirmed to have *EGFR*‐mutated LUAD.^2^ As a kind of transmembrane glycoprotein with tyrosine kinase activity, EGFR can activate signal pathways including PI3K/Akt/mTOR, RAS/Raf/MEK/ERK and STAT, and mediate the enhancement of LUAD malignant phenotype.^3^, ^4^ EGFR tyrosine kinase inhibitors (TKI) can competitively bind with ATP binding sites of EGFR, inactivate the activation of EGFR tyrosine kinases and downstream signaling pathways, and then inhibit the malignant phenotype of cells. At present, EGFR‐TKI is widely used in the treatment of *EGFR‐*sensitive mutation lung cancer patients, and significantly improves the prognosis of patients. However, most patients develop secondary resistance in different degrees within 12 months, which seriously limits the clinical efficacy of EGFR‐TKI.^5^ Therefore, it is of great significance to clarify the mechanism of secondary resistance for the treatment and drug discovery of LUAD. The activation of the c‐Met/PIK3R2 signal pathway to compensate for EGFR‐TKI anticancer effect is one of the main reasons for primary and secondary EGFR‐TKI resistance,^6^, ^7^, ^8^ but the activation mechanism of c‐Met/PIK3R2 signal pathway remains unclear.

Calpain 1 (CAPN1) belongs to the family of calcium dependent intracellular cysteine proteases, which is widely expressed in vivo. As one of the three major protein degradation systems, calpain plays a key role in cell proliferation, cytoskeleton remodeling, cell cycle regulation and apoptosis, glucose transport and cell signal transduction.^9^, ^10^, ^11^ Because of the extensive distribution and function, CAPN1 has been found to be abnormally expressed in pancreatic cancer, triple negative breast cancer, predictive pulmonary adenocarcinoma, ovarian cancer and gastric cancer, which is closely related to tumor progression and poor prognosis of patients.^12^, ^13^, ^14^, ^15^, ^16^ Meanwhile, increasing evidence in vitro and in vivo show that members of the calpain family can degrade different target proteins and affect the malignant phenotype of lung cancer cells,^17^, ^18^, ^19^ which suggests that CAPN1 may become a new target of clinical treatment and a potential prognostic indicator. However, the effect of CAPN1 on formation, progression, metastasis and EGFR‐TKI resistance of LUAD remains unclear. In this study, the potential relationship between CAPN1 and activation of c‐Met/PIK3R2 signaling pathway was found by bioinformatics analysis, and the exact position of CAPN1 in LUAD would be revealed.

Protein tyrosine phosphatase, nonreceptor type 1(PTPN1), a physiological target molecule of CAPN1,^20^, ^21^, ^22^ functionally targets multiple receptor tyrosine kinases (RTKs) for dephosphorylation, which is involved in the suppression of c‐Met related pathways. Some studies have shown that PTPN1 knockdown can improve the phosphorylation level of c‐Met, activate PI3K/Akt signal pathway, and then perform corresponding biological effects, including wound healing and the progress of ovarian cancer.^23^, ^24^, ^25^ Although the dephosphorylation ability of PTPN1 in tumors has been widely confirmed, contrasting findings have suggested that it can exert both cancer suppressing and promoting effects depending on the substrate involved and the cellular context.^26^, ^27^, ^28^ Therefore, the verification of PTPN1 effect is also essential to reveal the exact role of CAPN1 in LUAD.

In this study, we discovered a close relationship between the expression of CAPN1 and c‐Met/PIK3R2 and also revealed the abnormal expression and prognostic value of CAPN1 and PTPN1 in LUAD by bioinformatics analysis. Further, we confirmed the high expression of CAPN1 and low expression of PTPN1 in LUAD clinical tissues, and found the differential expression of CAPN1 and PTPN1 in EGFR wild‐type (WT) and mutant tissues, and then clarified the biological effect of CAPN1 and PTPN1, their relationship with EGFR‐TKI resistance, activation of c‐Met/PIK3R2 and the interaction with each other. These results demonstrated that CAPN1 and PTPN1 could be a potential therapeutic target for improving EGFR‐TKI sensitivity.

## Methods

### Clinical tissue specimens

We obtained 84 pairs of LUAD tissues and corresponding adjacent normal tissues after attaining informed consent from patients at the Second Xiangya Hospital, Central South University between September 2018 and June 2019. All patients' diagnoses were histopathologically confirmed. None of the patients had received any anticancer therapy prior to surgical resection. Tissues were snap‐frozen and stored in liquid nitrogen. This study was approved by the institutional research ethics committee.

### Cell culture and treatments

Cell lines

The human embryonic kidney cell 293T, human normal bronchial epithelial cells NHBE, human LUAD cell lines H1299, A549 and PC9, were procured from the American Tissue Culture Collection (ATCC). NHBE, H1299 and PC9 cells were cultured in RPMI‐1640 (Gibco, USA) with 10% fetal bovine serum (FBS, HyClone, Australia). 293T was cultured in DMEM high glucose (Gibco, USA) with 10% FBS, and A549 was cultured in DMEM/F12 (HyClone, Australia) with 10% FBS. To block protein synthesis of PTPN1, PC9 was treated with 10 μg/mL cycloheximide (CHX) for six or 12 hours.

### Induction of erlotinib resistance

PC9 cells were grown in 25 cm^2^ cell culture flasks and divided into PC9 erlotinib‐resistant (ER) and PC9 sensitive to erlotinib (Sen) groups. PC9 ER cells were treated with 0.2, 0.4, 0.8, 1.6, 3.2 and 6.4 μmol/L erlotinib gradiently for more than two weeks on each step until the cells grew stably in 6.4 μmol/L erlotinib for two weeks, and were then cultured in RPMI‐1640 + 10% FBS for two weeks. PC9 Sen cells were treated with isotonic PBS under the same conditions.

### Cell transfection

293T cells were cultured in a six‐well culture plates until the fusion degree was more than 60%. Then, 0.75 μg Gag‐pol, 0.30 μg Rev. and 0.45 μg VSV‐G plasmids were resuspended with 500 μL serum‐free DMEM with 1.5 μg objective plasmids including pLVX‐Puro Control, pLVX‐CAPN1, pLVX‐PTPN1 (Fenghbio, China), GV248‐Control and GV248‐shCAPN1 (Genechem, China) vector, respectively. Lipofectamine 2000 (Invitrogen, USA) 7.5 μL was mixed with the plasmids and incubated for 20 minutes. 293T cells were incubated by mixtures containing the plasmids for six hours with serum‐free DMEM, and then cultured by DMEM with 10% FBS for 48 hours. Cell supernatants as viral suspensions were filtered by 0.45 μm filter (Millipore, USA). A549 or PC9 cells were cultured in a six‐well culture plate until the fusion degree was about 60%, and RPMI‐1640 with 10% FBS blended viral suspensions in the ratio of 1:1 were applied to incubate A549 or PC9 cells for three days with 1 μg/mL polybrene (Sigma‐Aldrich, USA). Puromycin (Sigma‐Aldrich, USA) screened cells until there was a steady growth state.

### Quantitative real‐time PCR (qRT‐PCR)

The tissues were ground in mortar and the cells were digested by trypsin, and total RNA of cells or tissues were extracted by 1 mL RNAiso plus (Takara, Japan). The concentration of mRNA was detected by Nanodrop 2000 (Thermo Scientific, USA). Reverse transcription of mRNA was performed using a PrimeScript Strand cDNA synthesis kit (Takara, Japan). qRT‐PCR was performed to evaluate the mRNA expression levels by SYBR Premix Ex Taq II (Takara, Japan) on ABI Prism 7500 sequence detection system (Applied Biosystems, Life Technologies, USA). All groups were repeated at least three times independently. The expressions of mRNAs were calculated using the 2^−ΔΔCt^ method, and β‐Actin was regarded as internal reference. The sequences of β‐Actin primers: Forward 5'‐CCTGGCACCCAGCACAAT‐3′, Reverse 5'‐GCTGATCCACATCTGCT‐3′; CAPN1 primers: Forward 5'‐GGGTCCCAATTCCTCCAAGA‐3′, Reverse 5'‐CTGGAAATGGAAGATGCCGG‐3′; PTPN1 primers: Forward 5'‐TGCAGGATCAGTGGAAGGAG‐3′, Reverse 5'‐GTAGGGTGCGGCATTTAAGG‐3′; MET primers: Forward 5'‐ATGAGAGCTGCACCTTGACT‐3′, Reverse 5'‐CACCAGCCATAGGACCGTAT‐3′; PIK3R2 primers: Forward 5'‐GGGGACATTTCAAGGGAGGA‐3′, Reverse 5'‐CGTGGCGGTAGTGATTGATG‐3′.

### Immunohistochemistry

Cancer and normal adjacent tissues were collected to make paraffin sections. The paraffin sections were placed in an oven at a temperature of 67°C for two hours. After dewaxing and hydration, they were rinsed three times in PBS at pH7.4. Citrate solution (pH = 6.0) was added before it was placed in the microwave oven and heated to boiling. Paraffin sections were placed into the boiling buffer for 10 minutes, and rinsed twice with distilled water and PBS, respectively, for three minutes on each occasion. Then, 50 μL 3% H_2_O_2_ was added and incubated at the original temperature for 10 minutes, and rinsed three times with PBS, and the procedure was repeated three times. The sections were subsequently incubated with 50 μL of primary antibody for two hours, and rinsed three times with PBS. Then, 50 μL primary antibody enhancer was used to incubated sections for 20 minutes, and rinsed three times with PBS, and 50 μL enzyme‐labeled antimouse/rabbit polymer was used to incubated sections for 30 minutes, and rinsed three times with PBS. We then added 50 μL freshly prepared DAB solution to the sections. The sections were restained with hematoxylin, differentiated with 0.1% HCl, and rinsed with tap water to show the color of blue. They were dehydrated and dried with gradient alcohol. Xylene was used to render the sections transparent. The sections were sealed with neutral gum and observed after drying.

### Western blot

We used 1 mL RIPA buffer (Beyotime, China) to extract the total proteins of cells at 0°C for two hours, and centrifuged at 4°C, 15 000 r/minute. We collected the supernatant, trimmed the protein system and boiled for five minutes with loading buffer (Beyotime, China). Then, 10% SDS‐PAGE electrophoresis was used to separate proteins with different molecular weights and transferred to PVDF membrane (0.45 μm, Thermo Scientific), and 10% skimmed milk sealed the membranes for two hours. Diluting the primary antibody to the working concentration: anti‐CAPN1 (10538‐1‐AP, proteintech, USA) 1:500; anti‐PTPN1 (11334‐1‐AP, proteintech, USA) 1:1000; anti‐c‐Met (25869‐1‐AP, proteintech, USA) 1:500; PIK3R2(60225‐1‐Ig, proteintech, USA) 1:3000; antiphospho Met (ab73992, abcam, USA) 1:1000; anti‐phospho PIK3R2 (17366S, Cell Signaling Technology, USA) 1:1000. Second antibody incubated membranes for two hours at ordinary temperature. ECL western blotting substrate (Thermo Fisher, USA) was used for chemiluminescence. GelDoc XR+ instrument (Bio‐Rad, USA) was used to collect the luminescent signals.

### Coimmunoprecipitation (Co‐IP)

We cultured 293T cells in a 10 cm culture dish. After four generations expansion, the cells were rinsed with cold PBS and dissolved on ice for 15 minutes in the RIPA buffer with protease inhibitor. The lysate was centrifuged at 12 000 rpm for 10 minutes and the supernatant collected. Protein A/G MagBeads (Yeasen, China) and lysate were incubated at 4°C for one hour with rotation, and the magnetic frame was then used to remove the beads. The primary antibodies with working concentration were applied to incubate the supernatants for four hours, and incubated with Protein A/G MagBeads overnight. Then, MagBeads were placed in magnetic frame and washed five times with RIPA buffer. The binding protein was eluted with 2 x SDS loading buffer and boiled. The magnetic beads were then removed from the magnetic frame. Western blotting was used to detect the expression of precipitated PTPN1 protein.

### CCK‐8 assay

Cell proliferation

We inoculated cells in a 96‐well cell culture plate at 1 × 10^3^ cells/well overnight, and then 10 μL CCK‐8 reagent (Dojindo, Japan) was added to the plate and incubated for three hours without light. Absorbances were tested at 450 nm in 0, 24, 48, 72 and 96 hours by iMark Microplate light absorption reader (Bio‐Rad, USA). The relative proliferation abilities of cells were determined by the relative absorbances in 24, 48, 72 and 96 hours. All groups were repeated at least five times independently.

To detect the 50% inhibition concentration (IC_50_) to erlotinib in each group, we inoculated cells in a 96‐well cell culture plate at 5 × 10^3^ cells/well overnight, and these were then treated with 0, 0.125, 0.25, 0.5, 1, 2, 4, 8 μM erlotinib for 48 hours. Then, 10 μL CCK‐8 reagent was added to the plate and incubated for three hours without light. Absorbances were tested at 450 nm. Calculating IC_50_ to erlotinib (EIC_50_) value by fitting curve. All groups were repeated at least five times independently.

### Colony formation

We inoculated cells in a six‐well cell culture plate at 5 × 10^2^ cells/well for two weeks. The colon cells were rinsed three times with PBS and solidified with anhydrous ethanol for 10 minutes. Then, 0.1% crystal violet was used to dye the cells for 20 minutes, and the excess dye was washed away with tap water, and imageJ software (National Institutes of Health, USA) was used for colony counting.

### Transwell invasion and migration assay

Matrigel basement membrane matrix (BD Biosciences, USA) was diluted to 50 mg/L with serum‐free RPMI‐1640 and added into the transwell chamber (BD Biosciences, USA). After one hour at the original temperature, the remaining culture medium was removed. The cells were resuspended in serum‐free RPMI‐1640 containing BSA in preparation for cell suspension, and the density was adjusted to 2 × 10^5^ cells/mL. Then, 100 μL suspension was inoculated into the transwell chambers with or without Matrigel matrix (for invasion or migration), and 500 μL RPMI‐1640 containing 10% FBS was added to the lower chamber for routine culture for 24 hours. The nonpenetrating cells on the upper chamber surface were removed with a cotton swab. The cells were solidified using anhydrous ethanol for 10 minutes, and 0.1% crystal violet was used to dye them for 20 minutes; the excess dye was removed using PBS. There were five fields randomly in each well under 40 × 10 microscope (CX31, Olympus, Japan), and imageJ software was used for counting the cells.

### Statistical analysis

Data were expressed in mean ± SD and analyzed with the statistical software GraphPad Prism 8.0. A *t*‐test was used for comparison between the two groups, and one‐way ANOVA (Dunnett) was used for the comparison between multiple groups. *P* < 0.05 indicated statistical significance.

## Results

### Expression characteristics of CAPN1 and PTPN1 in LUAD from TCGA database

**Figure 1:**
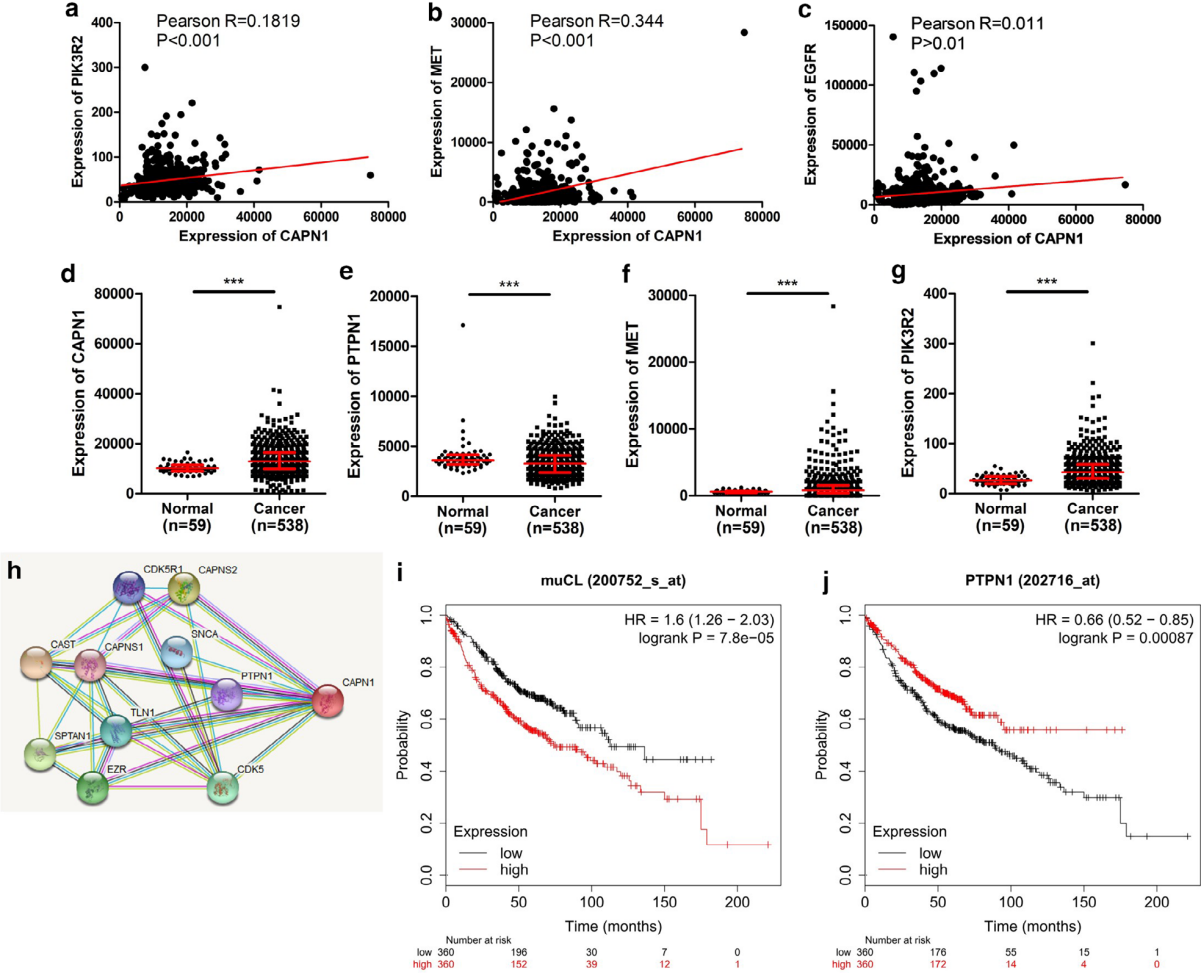
The expression of CAPN1/PTPN1 and its relationship with c‐Met/PIK3R2 in LUAD from TCGA database. The expressions of CAPN1/PTPN1 were anomalous and significantly correlated with c‐Met/PIK3R2 and prognosis of lung adenocarcinoma (LUAD) data from TCGA. (**a**, **b**, **c**) The correlation between CAPN1 and PIK3R2, MET or EGFR were analyzed by Pearson's correlation coefficient in the TCGA database. (**d**, **e**, **f**, **g**) Discrepant expressions of CAPN1, PTPN1, c‐Met and PIK3R2 in LUAD and normal tissues from TGCA database were measured by *t*‐test. *** *P* < 0.001. (**h**) PTPN1 was predicted as an interaction protein of CAPN1 (https://string‐db.org/). (**i**, **j**) The relationship between the expressions of CAPN1/PTPN1 and the prognosis of patients with LUAD were analyzed by the Kaplan‐Meier method through the database (http://kmplot.com/analysis/).

Owing to the fact that c‐Met/PIK3R2 pathway plays a pivotal role in the progress of LUAD as EGFR bypass signal, we screened the molecules closely related to the endogenous expression of c‐Met/PIK3R2 through the bioinformatics database (http://ualcan.path.uab.edu/index.html), and the results demonstrated that CAPN1 was positively correlated with the expression of endogenous c‐Met and PIK3R2 in LUAD (Fig 1a and b), but there was no significant correlation with EGFR expression (Fig 1c). However, as a proteolytic enzyme, CAPN1 may not directly affect c‐Met and PIK3R2 instead regulate c‐Met/PIK3R2 through intermediate molecules, which is dependent on the function of CAPN1. We then predicted (https://string-db.org/) and searched interaction molecules of CAPN1, and found an interaction protein PTPN1 (Fig 1h),^20^, ^21^, ^22^ which is involved in the dephosphorylation of various RTKs,^23^, ^24^, ^25^ so we speculated that CAPN1 regulates the activity of c‐Met/PIK3R2 pathway by degrading PTPN1. Based on the above conjecture, we analyzed the expression of CAPN1 and PTPN1 in LUAD, and found that CAPN1, c‐Met and PIK3R2 were highly expressed in LUAD (Fig 1d, f and g), Meanwhile, the relationships between CAPN1/PTPN1 and the prognosis of LUAD patients were analyzed through the database (http://kmplot.com/analysis/), and the results showed that the high expression of CAPN1 and the low expression of PTPN1 were related to the poor prognosis of patients (Fig 1i and j).

### Expression levels of CAPN1 and PTPN1 in LUAD clinical tissues and cell lines and their relationship with *EGFR*‐sensitive mutations

**Figure 2:**
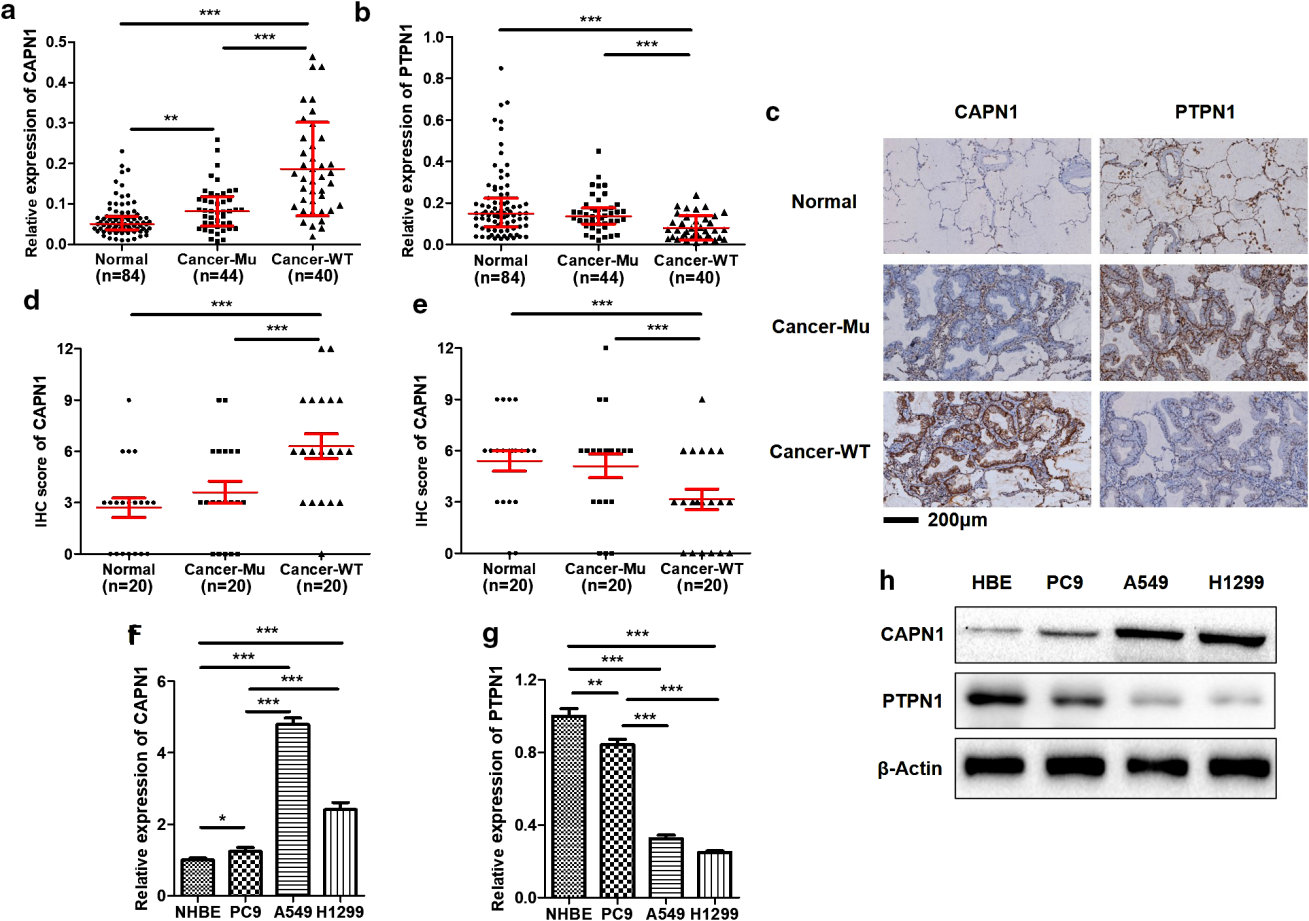
The expression of CAPN1/PTPN1 in *EGFR*‐mutant or nonmutant LUAD tissues and cells. The expressions of CAPN1/PTPN1 were differential and related to *EGFR*‐sensitive mutation in LUAD tissues and cells. (**a**, **b**) The mRNA expressions of CAPN1 and PTPN1 were detected by qRT‐PCR in normal, *EGFR*‐sensitive mutation and wild‐type (WT) *EGFR* tissues. ***P < 0.001. (**c**, **d**, **e**) The protein expressions of CAPN1 and PTPN1 were determined by immunohistochemistry (IHC). ** *P* < 0.001. (**f**, **g**) The mRNA expressions of CAPN1 and PTPN1 were measured by qRT‐PCR in human bronchial epithelial and LUAD cells. *P < 0.05, **P < 0.01, ***P < 0.001. (**h**) The protein expressions of CAPN1 and PTPN1 were tested via western blot in cell lines.

Based on the potential biological relationship between CAPN1/ PTPN1 and c‐Met/PIK3R2 and the significance of c‐Met/PIK3R2 bypass activation in EGFR‐TKI resistance, we detected the expression of CAPN1 and PTPN1 in clinical tissues from different sources. The results showed that CAPN1 mRNA and protein increased significantly in LUAD (Fig 2a, c and d), while PTPN1 decreased in LUAD (Fig 2b, c and e). Interestingly, we found that CAPN1 in wild‐type (WT) EGFR LUAD tissues was significantly higher than that in the activated *EGFR* mutation tissues (Fig 2a, c and d), and PTPN1 was lower, which was also observed in the cell models (Fig 2f, g and h). These results suggested that CAPN1/PTPN1 may play a biological role by regulating the c‐Met/PIK3R2 pathway in non‐*EGFR* mutation mediated LUAD, which may also be related to the primary resistance of EGFR‐TKI.

### Regulations of PTPN1 on c‐Met/PIK3R2 pathway and erlotinib sensitivity in LUAD cells

**Figure 3:**
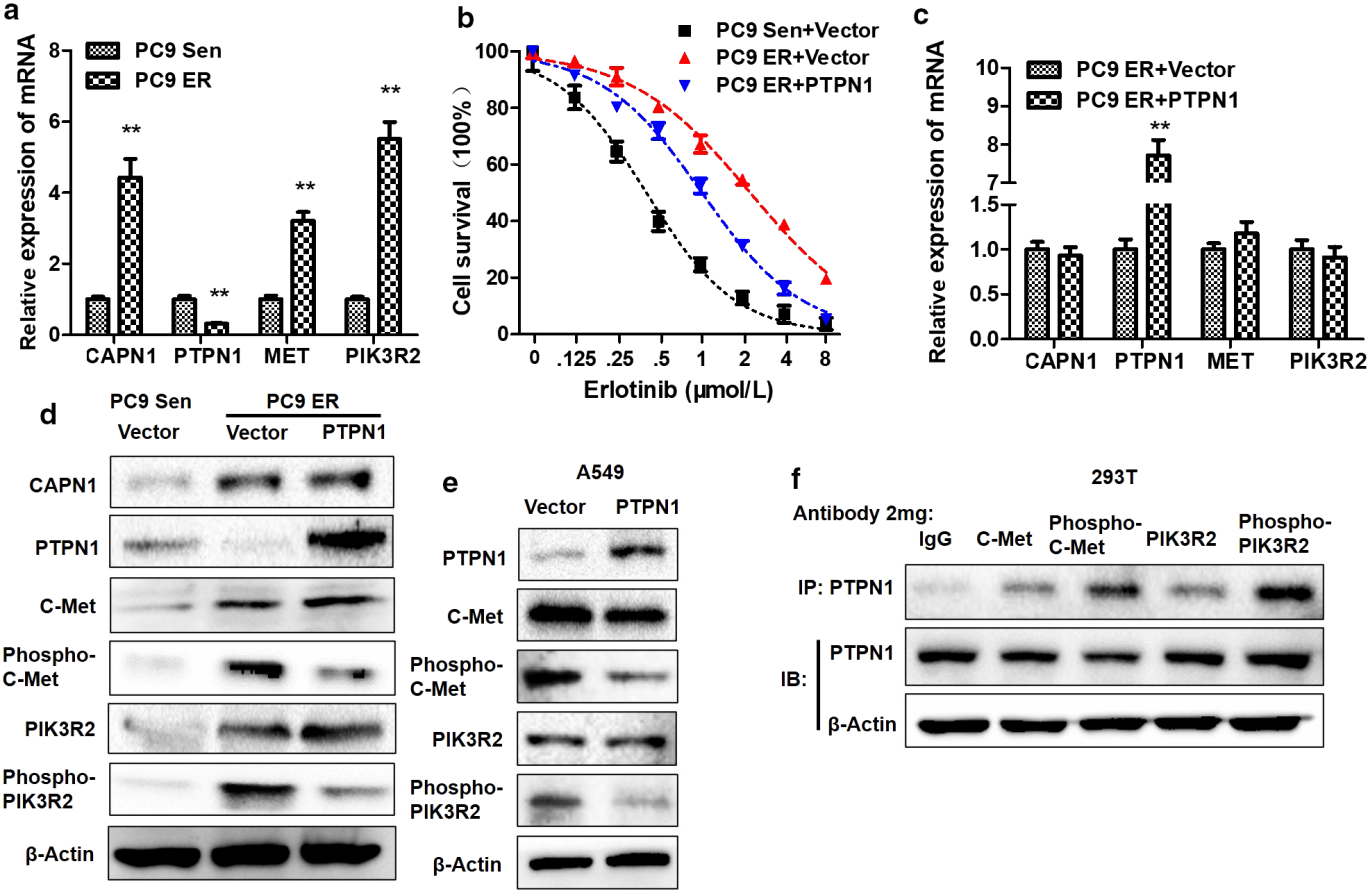
The regulations of PTPN1 on c‐Met/PIK3R2 and erlotinib resistance. PTPN1 could suppress erlotinib resistance (ER) and phosphorylation of c‐Met and PIK3R2 in PC9 ER cells. (**a**) The endogenous mRNA expressions of CAPN1, PTPN1, c‐Met and PIK3R2 were detected by qRT‐PCR in PC9 sensitive to erlotinib (PC9 Sen) and PC9 ER cells. **P < 0.001. (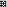), PC9 Sen; (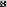), PC9 ER. (**b**) 50% inhibition concentrations to erlotinib (EIC_50_) were tested by CCK‐8 in PC9 Sen, PC9 ER and PC9 ER by overexpressing PTPN1 exogenously (PC9 ER + PTPN1). (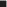), PC9 Sen+Vector; (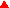), PC9 ER+Vector; (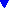), PC9 ER+PTPN1. (**c**) The mRNA expressions of CAPN1, PTPN1, c‐Met and PIK3R2 were detected by qRT‐PCR in PC9 ER and PC9 ER + PTPN1. **P < 0.001. (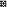), PC9 ER+Vector; (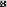), PC9 ER+PTPN1. (**d**, **e**) The protein expressions of CAPN1, PTPN1, c‐Met/PIK3R2 and phosphorylation of c‐Met/PIK3R2 were evaluated by western blot in PC9 ER and A549 with vector and PTPN1. (**f**) The combinations of PTPN1 with c‐Met, PIK3R2 and those phosphorylated were detected by Co‐IP assay.

In order to understand the regulatory effect of PTPN1 on c‐Met/PIK3R2 pathway and erlotinib sensitivity in LUAD, we first constructed erlotinib resistant in PC9 cells which are *EGFR*‐sensitive mutations, and then found that erlotinib resistance could mediate the upregulation of CAPN1 expression and c‐Met/PIK3R2 phosphorylation and the downregulation of PTPN1 expression (Fig 3a and d), which indicated that CAPN1 and PTPN1 may be involved in the formation of erlotinib resistance. Therefore, we overexpressed PTPN1 exogenously in PC9 ER cells, and found that PTPN1 could significantly reduce EIC_50_ of PC9 ER cells (Fig 3b). In order to further reveal the effects of PTPN1 on the c‐Met/PIK3R2 pathway, using qRT‐PCR and western blot, we found that PTPN1 could significantly inhibit the phosphorylation level of c‐Met and PIK3R2 in A549 and PC9 ER cells (Fig 3d and e), but had no significant effect on mRNA (Fig 3c) and total protein (Fig 3d and e). The results of Co‐IP showed that PTPN1 was combined with total and phosphorylated c‐Met/PIK3R2 (Fig 3f).

### Effects of PTPN1 on LUAD cell proliferation and metastasis in vitro

**Figure 4:**
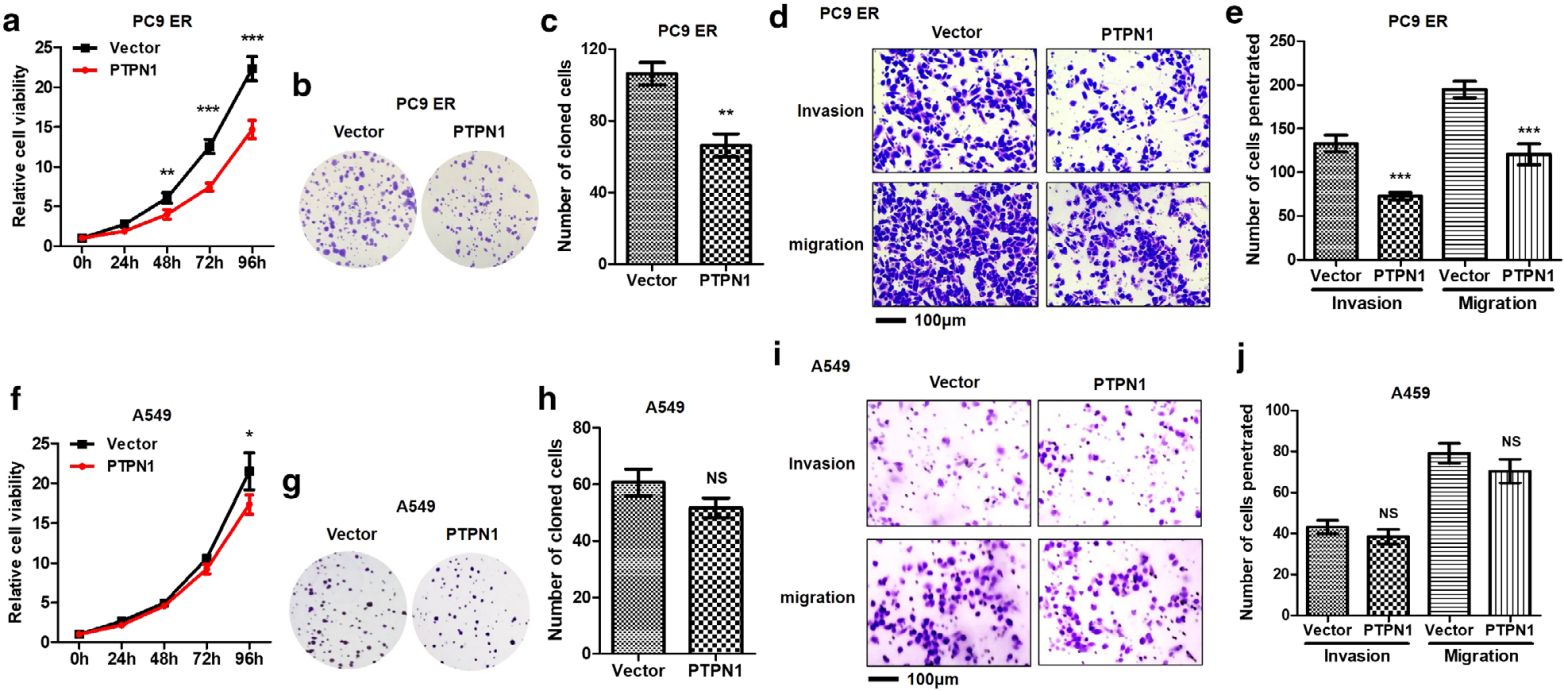
The effects of PTPN1 on malignant phenotype of PC9 ER and A549 cells. PTPN1 inhibited the proliferation and metastasis of PC9 ER and A549 cells. (**a**) Relative proliferative capacities of PC9 ER vector and PTPN1 were tested by CCK‐8 assay. **P < 0.01. ***P < 0.001. (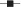), Vector; (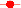), PTPN1. (**b**, **c**) Colony formation abilities of PC9 ER vector and PTPN1 were measured by plate colony formation assay. **P < 0.01. (**d**, **e**) Metastasis abilities of PC9 ER vector and PTPN1 were estimated by transwell migration and invasion assays. ***P < 0.001. (**f**) Relative proliferative capacities of A549 vector and PTPN1 were tested by CCK‐8 assay. *P < 0.05. (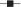), Vector; (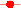), PTPN1. (**g**, **h**) Colony formation abilities of A549 vector and PTPN1 were measured by plate colony formation assay. NS, not significant. (**i**, **j**) Metastasis abilities of A549 vector and PTPN1 were estimated by transwell migration and invasion assays. NS, not significant.

Although PTPN1 has been shown to play a certain role in promoting lung cancer,^27^ this conclusion is contrary to the existing prediction of bioinformation and experimental results, so we reverified the biological effect of PTPN1. The results showed that cell proliferation (Fig 4a) and clonogenesis (Fig 4b and c) were significantly weakened after overexpression of PTPN1 in PC9 ER cell lines. Moreover, the number of invasion and metastasis cells decreased significantly (Fig 4d and e). Based on the fact that the role of PTPN1 in tumor is still controversial,^28^ we speculated that the reason for the above paradox was due to the dramatic changes of gene expression profile between EGFR‐TKI sensitivity and resistance, which leads to the malignant behavior of cells mediated by differential molecules, and then causes the discrepant effects of PTPN1. In order to prove this phenomenon from the negative side, we further analyzed the effect of overexpressing PTPN1 on the malignant phenotype of *EGFR* wild‐type lung cancer cell A549, whose results showed that the inhibitory effect of PTPN1 on malignant phenotype of A549 were not remarkable compared with those in PC9 ER (Fig 4f, g, h, i and j). Although this phenomenon was contrary to the effects that PTPN1 suppressing phosphorylation of c‐Met/PIK3R2, considering the extensive inhibitory effect of PTPN1 on phosphorylation, we speculated that PTPN1 might act on other pivotal targets in A549 cells, which led to our contradictory observation from a limited perspective.

### Regulations of CAPN1 on PTPN1/c‐Met/PIK3R2 pathway and erlotinib sensitivity in LUAD cells

**Figure 5:**
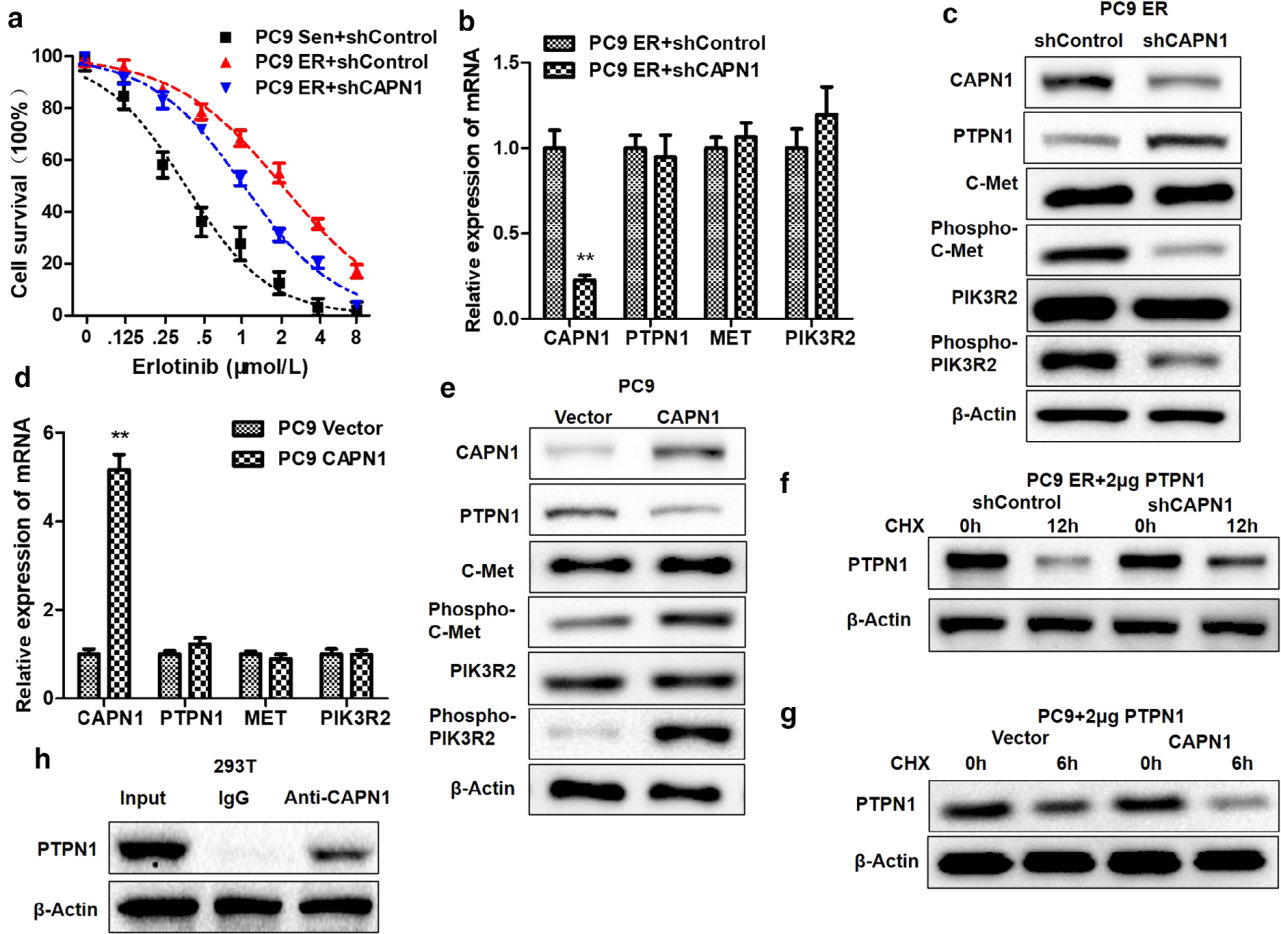
The regulations of CAPN1 on PTPN1, c‐Met/PIK3R2 and erlotinib resistance. CAPN1 was closely related to erlotinib resistance (ER) and phosphorylation of c‐Met and PIK3R2 in human LUAD cell PC9 with *EGFR* mutation. (**a**) EIC_50_ values of PC9 Sen, PC9 ER and PC9 ER with the knockdown of CAPN1 by short hairpin RNA (shRNA) (PC9 ER + shCAPN1) were evaluated by CCK‐8 assay. (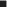), PC9 Sen+shControl; (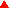), PC9 ER+shControl; (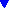), PC9 ER+shCAPN1. (**b**, **c**) The mRNA and protein expressions of CAPN1, PTPN1, c‐Met and PIK3R2 were detected by qRT‐PCR and western blot in PC9 ER and PC9 ER + shCAPN1. **P < 0.001. (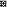), PC9 ER+shControl; (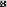), PC9 ER+shCAPN1. (**d**, **e**) The mRNA and protein expressions of CAPN1, PTPN1, c‐Met and PIK3R2 were detected by qRT‐PCR and western blot in PC9 vector and PC9 CAPN1. **P < 0.001. (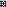), Vector; (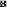), PTPN1. (**f**, **g**) The degradation rates of PTPN1 in PC9 ER and PC9 ER + shCAPN1 or PC9 vector and PC9 CAPN1 were detected by western blot in cases of cycloheximide (CHX) treatment. (**h**) The combination of CAPN1 and PTPN1 was verified by Co‐IP assay.

Because of the remarkable upregulation of CAPN1 in PC9 ER cell lines, it is essential to identify the effect of CAPN1 in LUAD. We used shRNA to interfere with CAPN1 in PC9 ER, and found that EIC50 was significantly reduced (Fig 5a). At the same time, the results of qRT‐PCR and western blot indicated that interfering with CAPN1 could significantly inhibit the phosphorylation of c‐Met/PIK3R2 (Fig 5c), but had no significant effect on mRNA and total protein (Fig 5b and c). Similarly, we found that the phosphorylation levels of c‐Met and PIK3R2 increased after overexpression of CAPN1 exogenously, while the mRNA and total c‐Met and PIK3R2 did not change significantly (Fig 5d and e). The regulation of CAPN1 on PTPN1 has been found in biological prediction and a macrophage model,^20^, ^21^, ^22^ but the regulation in LUAD still needs to be clarified. We observed the effect of CAPN1 on the degradation rate of PTPN1 through inhibiting the synthesis of total protein mediated by CHX, and the results showed that interference with CAPN1 could significantly inhibit the degradation of PTPN1 (Fig 5f), while overexpression of CAPN1 could significantly promote the degradation of PTPN1 (Fig 5g). Co‐IP assay confirmed the interaction between CAPN1 and PTPN1 (Fig 5h).

### Effects of CAPN1 and rescued by PTPN1 on LUAD cell proliferation and metastasis in vitro

**Figure 6:**
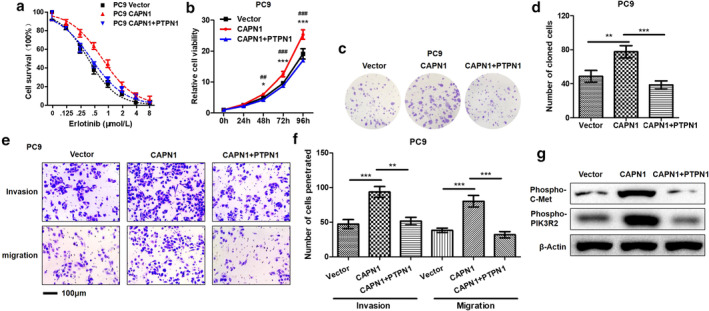
The effects of CAPN1 and PTPN1 rescued on malignant phenotype and c‐Met/PIK3R2 of PC9 cells. There are facilitations of CAPN1 on ER, proliferation and metastasis of PC9 cells, which could be antagonized by PTPN1. (**a**, **b**) EIC_50_ values and relative proliferative capacities of PC9 vector, CAPN1 and CAPN1 + PTPN1 cells were measured by CCK‐8. Compared with PC9 vector, PC9 CAPN1 cells was *P < 0.05 or ***P < 0.001. Compared with PC9 CAPN1, PC9 CAPN1 + PTPN1 cells was ^##^ *P* < 0.05 or ^###^ *P* < 0.001. (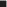), PC9 Vector; (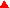), PC9 CAPN1; (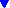), PC9 CAPN1+PTPN1. (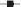), Vector; (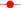), CAPN1; (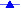), CAPN1+PTPN1. (**c**, **d**) Colony formation abilities of PC9 Vector, CAPN1 and CAPN1 + PTPN1 cells were evaluated by plate colony formation assay. **P < 0.01. ***P < 0.001. (**e**, **f**) Metastasis abilities of PC9 vector, CAPN1 and CAPN1 + PTPN1 cells were tested by transwell migration and invasion assays. **P < 0.01. ***P < 0.001. (**g**) Phosphorylation of c‐Met and PIK3R2 were tested via western blot in PC9 vector, CAPN1 and CAPN1 + PTPN1 cells.

All the above results suggested that CAPN1/PTPN1 may be involved in promoting EGFR‐TKI resistance and malignant behavior of LUAD, so we overexpressed CAPN1 and rescued PTPN1 in PC9 WT cells, and found that overexpression of CAPN1 could enhance erlotinib resistance, while PTPN1 could make PC9 cells re‐sensitive to erlotinib (Fig 6a). Similarly, CAPN1 could strengthen the proliferation and clonogenesis ability of PC9 cells in vitro (Fig 6b, c and d), enhance metastasis (Fig 6e and f) and promote phosphorylation of c‐Met and PIK3R2 (Fig 6g). Meanwhile, the rescue of PTPN1 could inhibit the malignant phenotype and phosphorylation of c‐Met and PIK3R2 mediated by CAPN1.

## Discussion

The activation of c‐Met/PIK3R2 pathway not only mediates the enhancement of malignant phenotype,^29^, ^30^ but also leads to EGFR‐TKI resistance,^6^, ^7^, ^8^, ^31^ which is one of the pivots in LUAD development. It is essential for cancer treatment and management to clarify the regulatory mechanism of c‐Met/PIK3R2. To determine the potential core molecules regulating c‐Met/PIK3R2, we screened out CAPN1 which is closely related to endogenous c‐Met/PIK3R2 expressions by bioinformatics methods as the target molecule of our research. Previous studies have revealed that CAPN1 is dysregulated in multiple malignancies. For example, CAPN1 has been reported to be an independent marker for poor relapse‐free survival in breast cancer patients treated with trastuzumab,^32^ and inhibition of CAPN1 could attenuate cisplatin‐induced apoptosis in triple‐negative breast cancer (TNBC) cells,^33^ moreover, CAPN1 is positively correlated with lymph node status of TNBC patients, suggesting its role as a prognostic factor.^13^ CAPN1 was overexpressed in pancreatic cancer (PC) tissues and cells and associated with tumor site, metastasis, TNM stage and overall survival of PC patients.^12^ In addition, CAPN1 could promote AKT signaling and melanoma cell growth via degrading tumor suppressor NF1, and combination treatment of CAPN1 inhibitor and with MEKi trametinib could inhibit cell malignant phenotype more effectively compared to treatment with trametinib alone,^34^ which also indicated that CAPN1 can be a potential target for tumor treatment. Clinical studies have shown that the expression of CAPN1 is positively related to tumor volume, invasion, distant metastasis and poor prognosis in patients with laryngeal cancer. In addition, CAPN1 is related to the increased level of ERK phosphorylation,^35^ which also suggests the potential connection between CAPN1 and the activation of proto‐oncogene phosphorylation. These studies indicated that CAPN1 affect a variety of malignant tumors as a tumor promoter, and these evidences pointed to the effects of CAPN1 on drug resistance and molecular phosphorylation activity, which arouses our interest in the question of what role CAPN1 plays in LUAD EGFR‐TKI and malignant behavior. Therefore, we spotted that there is a potential target molecule PTPN1 in CAPN1, which is related to dephosphorylation of manifold RTKs,^23^, ^24^, ^25^ but there is little evidence for the interaction between CAPN1 and PTPN1 in cancer, and the effects of PTPN1 on cancer is still controversial.

PTPN1, as a member of protein tyrosine phosphatases (PTPs), maintains cellular homeostasis via participation in the balanced action of protein tyrosine kinases (PTKs) and PTPs. Recently, that has been an increasing number of reports which have demonstrated that PTPN1 is involved in human disorders such as diabetes, obesity, and cancer.^36^, ^37^, ^38^ However, different research results have demonstrated that PTPN1 plays a dual role in malignant tumors, which depends on the substrate expression level of PTPN1 in distinct cells.^28^ For example, PTPN1 were inactivated in acute lymphoblastic leukemia SupB15 cells with resistance of ABL kinase inhibitor STI571, and accompanied by phosphorylating Bcr‐Abl, and exogenous inhibition of PTPN1 could attenuate STI571‐induced apoptosis, and enhance the resistance to STI571, which suggested that PTPN1 may be closely related to the sensitivity of targeted drugs. However, in the chronic lymphocytic leukemia cell model, PTPN1 could disrupt the degradation of Bcr‐Abl protein mediated by synthetic steroidal glycoside SBF‐1 via the lysosome pathway, which leads to the resistance of K562 cells to imatinib.^39^ PTPN1 also had double effects in glioma. Akasaki *et al*.^40^ found that troglitazone could inactivate STAT3 and Bcl‐2 by activating PTPN1, thereby promoting apoptosis mediated by caspase systems, making cells sensitive to chemotherapy drugs. In contrast, Jin *et al*.^41^ declared that PTPN1 was highly expressed in glioma tissue, and promoted the progression of gliomas by activating the MAPK/ERK and PI3K/Akt. These reports show that PTPN1 plays a diverse role in cells with discrepant molecular backgrounds. In addition, Sangwan *et al*.^42^ pointed out that loss of PTPN1 and T cell phosphatase (TCPTP) could mediate the phosphorylated activation of c‐Met in mouse model and human cervical cancer cells Hela, and then promote the metastasis of tumor cells, and confirmed the interaction between PTPN1 and c‐Met in vitro. Nievergall *et al*.^43^ found that abrogated PTPN1 causes enhanced and prologed erythropoietin‐producing hepatocellular receptor (Eph)A3 phosphorylation and biological function. Moreover, Krishnan *et al*.^44^ pointed out that the endogenous hydrogen sulfide could phosphorylate ERK protein via suppression of PTPN1. These studies indicate that PTPN1 is related to the dephosphorylation and inactivation of tumor associated proteins.

On account of potential values of CAPN1/PTPN1 on progression and EGFR‐TKI resistance of LUAD, we intended to reveal the expression characteristics and biological functions of CAPN1/PTPN1 in LUAD. First, through bioinformatics methods, CAPN1 was discovered to be significantly correlated with endogenous c‐Met and PIK3R2 instead of EGFR, and overexpressed in LUAD, while its interactor PTPN1 was downregulated in LUAD. Kaplan‐Meier analysis demonstrated higher CAPN1 and lower PTPN1 is related to poor overall survival in patients with LUAD. The results of qRT‐PCR and IHC are consistent with bioinformatics results; meanwhile, the expressions of CAPN1/PTPN1 showed a significant difference between tissues and cells with WT and mutated *EGFR*, which indicated that the carcinogenesis compensation of WT EGFR was bound up with superior CAPN1 and inferior PTPN1. Therefore, we speculated that CAPN1/PTPN1 plays a crucial role in EGFR‐TKI resistance and cell malignant phenotypes by activating EGFR bypass signal c‐Met/PIK3R2. Next, we found that PTPN1 was downregulated in ER cells, which suggested PTPN1 may participate in ER of LUAD. Overexpression of PTPN1 could suppress the phosphorylation of c‐Met and PIK3R2, enhance the sensitiveness to erlotinib, and inhibit cell proliferation and metastasis in PC9 ER, instead in A549. Although these trends were not consistent with previous research results,^27^ it is probably due to the difference of gene expression profile between ER cells and WT cells. On the other hand, we observed that downregulating CAPN1 by shRNA could reverse erlotinib resistance, dephosphorylate c‐Met/PIK3R2 and prolong PTPN1 expression in PC9 ER cells, while augmenting CAPN1 could enhance erlotinib resistance, phosphorylated c‐Met/PIK3R2 and accelerate the degradation of PTPN1 in PC9 cells, as well as strengthen cell proliferation and metastasis, but these effects could be antagonized by upregulating PTPN1. These results indicated that CAPN1/PTPN1, as novel regulatory factors of c‐Met/PIK3R2, are involved in cell proliferation, metastasis and EGFR‐TKI resistance in LUAD, but the binding sites of molecular interaction and vivo functions still need to be defined. We will further evaluate the exact value of CAPN1/PTPN1 in the diagnostic markers and treatment targets of LUAD through animal models and molecular biological experiments to provide a new basis for the clinical study of LUAD.

## Disclosure

The authors have no conflicts of interest to declare.

## References

[1] Bray F , Ferlay J , Soerjomataram I , Siegel RL , Torre LA , Jemal A . Global cancer statistics 2018:GLOBOCAN estimates of incidence and mortality worldwide for 36 cancers in 185 countries. CA Cancer J Clin 2018; 68: 394–424. 30207593 10.3322/caac.21492

[2] Shi Y , Joseph Siu‐Kie A , Thongprasert S *et al* A prospective, molecular epidemiology study of EGFR mutations in Asian patients with advanced non–small‐cell lung cancer of adenocarcinoma histology (PIONEER). J Thorac Oncol 2014; 9: 154–62. 24419411 10.1097/JTO.0000000000000033 PMC4132036

[3] Clayton SJ , Scott FM , Walker J *et al* K‐ras point mutation detection in lung cancer: Comparison of two approaches to somatic mutation detection using ARMS allele‐specific amplification. Clin Chem 2000; 46: 1929–38. 11106325

[4] Zhang Y , Xiang C , Wang Y *et al* lncRNA LINC00152 knockdown had effects to suppress biological activity of lung cancer via EGFR/PI3K/AKT pathway. Biomed Pharmacother 2017; 94: 644–51. 28787699 10.1016/j.biopha.2017.07.120

[5] Sheng Z , Zhang Y . The efficacy of epidermal growth factor receptor tyrosine kinase inhibitors in non–small cell lung cancer harboring wild‐type epidermal growth factor receptor: A meta‐analysis of 25 RCTs. Am J Clin Oncol 2017; 40: 362–9. 25647830 10.1097/COC.0000000000000179

[6] Nanjo S , Arai S , Wang W . MET copy number gain is associated with Gefitinib resistance in Leptomeningeal Carcinomatosis of EGFR‐mutant lung cancer. Mol Cancer Ther 2017; 16: 506–15. 28138027 10.1158/1535-7163.MCT-16-0522

[7] Wu H , Fan F , Liu Z , Shen C , Wang A , Lu Y . Norcantharidin combined with EGFR‐TKIs overcomes HGF‐induced resistance to EGFR‐TKIs in EGFR mutant lung cancer cells via inhibition of met/PI3k/Akt pathway. Cancer Chemother Pharm 2015; 76: 307–15. 10.1007/s00280-015-2792-x 26063323

[8] Moores SL , Chiu ML , Bushey BS . A novel Bispecific antibody targeting EGFR and cMet is effective against EGFR inhibitor‐resistant lung tumors. Cancer Res 2016; 76: 3942–53. 27216193 10.1158/0008-5472.CAN-15-2833

[9] Tadic V , Klein C , Hinrichs F , Münchau A , Lohmann K , Brüggemann N . CAPN1 mutations are associated with a syndrome of combined spasticity and ataxia. J Neurol 2017; 264: 1008–10. 28321562 10.1007/s00415-017-8464-5

[10] Miller DJ , Adams SE , Hallett MB , Allemann RK . Calpain‐1 inhibitors for selective treatment of rheumatoid arthritis: What is the future? Future Med Chem 2013; 5: 2057–74. 24215346 10.4155/fmc.13.172

[11] Ono Y , Saido TC , Sorimachi H . Calpain research for drug discovery: Challenges and potential. Nat Rev Drug Discov 2016; 15: 854–76. 27833121 10.1038/nrd.2016.212

[12] Yu LM , Zhu YS , Xu CZ , Zhou LL , Xue ZX , Cai ZZ . High calpain‐1 expression predicts a poor clinical outcome and contributes to tumor progression in pancreatic cancer patients. Clin Transl Oncol 2019; 21: 924–32. 30565085 10.1007/s12094-018-02006-6

[13] Al‐Bahlani SM , Al‐Rashdi RM , Kumar S , Al‐Sinawi SS , Al‐Bahri MA , Shalaby AA . Calpain‐1 expression in triple‐negative breast cancer: A potential prognostic factor independent of the proliferative/apoptotic index. Biomed Res Int 2017; 2017: 9290425. 28536704 10.1155/2017/9290425 PMC5425834

[14] Ruppert AM , Baud L , Rabbe N *et al* Calpain 1 in bronchoalveolar lavage fluid is associated with poor prognosis in lepidic predominant pulmonary adenocarcinoma. Bull Cancer 2019; 106: 179–88. 30683309 10.1016/j.bulcan.2018.11.014

[15] Zhang S , Deen S , Storr SJ *et al* Calpain system protein expression and activity in ovarian cancer. J Cancer Res Clin Oncol 2019; 145: 345–61. 30448882 10.1007/s00432-018-2794-2 PMC6373250

[16] Liu B , Zhou Y , Lu D *et al* Comparison of the protein expression of calpain‐1, calpain‐2, calpastatin and calmodulin between gastric cancer and normal gastric mucosa. Oncol Lett 2017; 14: 3705–10. 28927135 10.3892/ol.2017.6617 PMC5588046

[17] Xu F , Gu J , Lu C *et al* Calpain‐2 enhances non‐small cell lung cancer progression and Chemoresistance to paclitaxel via EGFR‐pAKT pathway. Int J Biol Sci 2019; 15: 127–37. 30662353 10.7150/ijbs.28834 PMC6329934

[18] Gu J , Xu FK , Zhao GY *et al* Capn4 promotes non‐small cell lung cancer progression via upregulation of matrix metalloproteinase 2. Med Oncol 2015; 32: 51. 25636510 10.1007/s12032-015-0500-7

[19] Lau JK , Brown KC , Dom AM *et al* Capsaicin induces apoptosis in human small cell lung cancer via the TRPV6 receptor and the calpain pathway. Apoptosis 2014; 19: 1190–201. 24878626 10.1007/s10495-014-1007-y PMC4072851

[20] Lin YW , Lee B , Liu PS , Wei LN . Receptor‐interacting protein 140 orchestrates the dynamics of macrophage M1/M2 polarization. J Innate Immun 2016; 8: 97–107. 26228026 10.1159/000433539 PMC5105833

[21] Kuchay SM , Kim N , Grunz EA , Fay WP , Chishti AH . Double knockouts reveal that protein tyrosine phosphatase 1B is a physiological target of calpain‐1 in platelets. Mol Cell Biol 2007; 27: 6038–52. 17576811 10.1128/MCB.00522-07 PMC1952154

[22] Kuchay SM , Wieschhaus AJ , Marinkovic M , Herman IM , Chishti AH . Targeted gene inactivation reveals a functional role of calpain‐1 in platelet spreading. J Thromb Haemost 2012; 10: 1120–32. 22458296 10.1111/j.1538-7836.2012.04715.x PMC3956748

[23] Kakazu A , Sharma G , Bazan HE . Association of protein tyrosine phosphatases (PTPs)‐1B with c‐met receptor and modulation of corneal epithelial wound healing. Invest Ophthalmol Vis Sci 2008; 49: 2927–35. 18579758 10.1167/iovs.07-0709 PMC2556234

[24] Sangwan V , Abella J , Lai A *et al* Protein‐tyrosine phosphatase 1B modulates early endosome fusion and trafficking of met and epidermal growth factor receptors. J Biol Chem 2011; 286: 45000–13. 22045810 10.1074/jbc.M111.270934 PMC3247994

[25] Zhang J , Wang Z , Zhang S *et al* Spatial regulation of signaling by the coordinated action of the protein tyrosine kinases MET and FER. Cell Signal 2018; 50: 100–10. 29920310 10.1016/j.cellsig.2018.06.006 PMC6530786

[26] Martínez‐Meza S , Díaz J , Sandoval‐Bórquez A *et al* AT2 receptor mediated activation of the tyrosine phosphatase PTP1B blocks Caveolin‐1 enhanced migration, invasion and metastasis of cancer cells. Cancers 2019; 11: E1299. 31484460 10.3390/cancers11091299 PMC6770525

[27] Liu H , Wu Y , Zhu S *et al* PTP1B promotes cell proliferation and metastasis through activating src and ERK1/2 in non‐small cell lung cancer. Cancer Lett 2015; 359: 218–25. 25617799 10.1016/j.canlet.2015.01.020

[28] Lessard L , Stuible M , Tremblay ML . The two faces of PTP1B in cancer. Biochim Biophys Acta 1804; 2010: 613–9. 10.1016/j.bbapap.2009.09.018 19782770

[29] Pasquini G , Giaccone G . C‐MET inhibitors for advanced non‐small cell lung cancer. Expert Opin Investig Drugs 2018; 27: 363–75. 10.1080/13543784.2018.1462336 29621416

[30] Song L , Li D , Gu Y *et al* MicroRNA‐126 targeting PIK3R2 inhibits NSCLC A549 cell proliferation, migration, and invasion by regulation of PTEN/PI3K/AKT pathway. Clin Lung Cancer 2016; 17: e65–75. 27236384 10.1016/j.cllc.2016.03.012

[31] Wang Q , Yang S , Wang K , Sun SY . MET inhibitors for targeted therapy of EGFR TKI‐resistant lung cancer. J Hematol Oncol 2019; 12: 63. 31227004 10.1186/s13045-019-0759-9 PMC6588884

[32] Pu X , Storr SJ , Ahmad NS *et al* Calpain‐1 is associated with adverse relapse free survival in breast cancer: A confirmatory study. Histopathology 2016; 68: 1021–9. 26496999 10.1111/his.12896

[33] Al‐Bahlani SM , Al‐Bulushi KH , Al‐Alawi ZM , Al‐Abri NY , Al‐Hadidi ZR , Al‐Rawahi SS . Cisplatin induces apoptosis through the endoplasmic reticulum‐mediated, Calpain 1 pathway in triple‐negative breast cancer cells. Clin Breast Cancer 2017; 17: e103–12. 28089626 10.1016/j.clbc.2016.12.001

[34] Alon M , Arafeh R , Lee JS *et al* CAPN1 is a novel binding partner and regulator of the tumor suppressor NF1 in melanoma. Oncotarget 2018; 9: 31264–77. 30131853 10.18632/oncotarget.25805 PMC6101293

[35] Starska K , Forma E , Jóźwiak P *et al* Gene/protein expression of CAPN1/2‐CAST system members is associated with ERK1/2 kinases activity as well as progression and clinical outcome in human laryngeal cancer. Tumour Biol 2016; 37: 13185–203. 27456359 10.1007/s13277-016-5178-8

[36] Huang Q , Han L , Liu Y *et al* Elevation of PTPN1 promoter methylation is a significant risk factor of type 2 diabetes in the Chinese population. Exp Ther Med 2017; 14: 2976–82. 29042909 10.3892/etm.2017.4924 PMC5639402

[37] Huang S , Liu L , Xiang Y *et al* Association of PTPN1 polymorphisms with breast cancer risk: A case‐control study in Chinese females. J Cell Biochem 2019; 120: 1–12. 10.1002/jcb.28490 30805963

[38] Koyama N , Koschmieder S , Tyagi S *et al* Inhibition of phosphotyrosine phosphatase 1B causes resistance in BCR‐ABL‐positive leukemia cells to the ABL kinase inhibitor STI571. Clin Cancer Res 2006; 12: 2025–31. 16609011 10.1158/1078-0432.CCR-04-2392

[39] Elgehama A , Chen W , Pang J *et al* Blockade of the interaction between Bcr‐Abl and PTB1B by small molecule SBF‐1 to overcome imatinib‐resistance of chronic myeloid leukemia cells. Cancer Lett 2016; 372: 82–8. 26721204 10.1016/j.canlet.2015.12.014

[40] Akasaki Y , Liu G , Matundan HH *et al* A peroxisome proliferator‐activated receptor‐gamma agonist, troglitazone, facilitates caspase‐8 and ‐9 activities by increasing the enzymatic activity of protein‐tyrosine phosphatase‐1B on human glioma cells. J Biol Chem 2006; 281: 6165–74. 16319070 10.1074/jbc.M505266200

[41] Jin T , Li D , Yang T , Liu F , Kong J , Zhou Y . PTPN1 promotes the progression of glioma by activating the MAPK/ERK and PI3K/AKT pathways and is associated with poor patient survival. Oncol Rep 2019; 42: 717–25. 31173266 10.3892/or.2019.7180

[42] Sangwan V , Paliouras GN , Abella JV *et al* Regulation of the met receptor‐tyrosine kinase by the protein‐tyrosine phosphatase 1B and T‐cell phosphatase. J Biol Chem 2008; 283: 34374–83. 18819921 10.1074/jbc.M805916200 PMC2662243

[43] Nievergall E , Janes PW , Stegmayer C *et al* PTP1B regulates Eph receptor function and trafficking. J Cell Biol 2010; 191: 1189–203. 21135139 10.1083/jcb.201005035 PMC3002030

[44] Krishnan N , Fu C , Pappin DJ , Tonks NK . H2s‐induced sulfhydration of the phosphatase PTP1B and its role in the endoplasmic reticulum stress response. Sci Signal 2011; 4: ra86. 22169477 10.1126/scisignal.2002329 PMC3328411

